# Benefits, Harms, and Stakeholder Perspectives Regarding Opioid Therapy for Pain in Individuals With Metastatic Cancer: Protocol for a Descriptive Cohort Study

**DOI:** 10.2196/54953

**Published:** 2024-03-13

**Authors:** Katie Fitzgerald Jones, Gretchen White, Antonia Bennett, Hailey Bulls, Paula Escott, Sarah Orris, Elizabeth Escott, Stacy Fischer, Megan Hamm, Tamar Krishnamurti, Risa Wong, Thomas W LeBlanc, Jane Liebschutz, Salimah Meghani, Cardinale Smith, Jennifer Temel, Christine Ritchie, Jessica S Merlin

**Affiliations:** 1 New England Geriatrics Research, Education, and Clinical Center (GRECC) Jamaica Plain, MA United States; 2 University of Pittsburgh Pittsburgh, PA United States; 3 University of North Carolina Chapel Hill, NC United States; 4 University of Colorado Aurora, CO United States; 5 Duke University School of Medicine Durham, NC United States; 6 University of Pennsylvania Philadelphia, PA United States; 7 Icahn School of Medicine at Mount Sinai New York, NY United States; 8 Division of Hematology/Oncology, Massachusetts General Hospital Boston, MA United States; 9 Center for Aging and Serious Illness Massachusetts General Hospital Boston, MA United States; 10 Department of Medicine Harvard Medical School Boston, MA United States

**Keywords:** cancer, cancer-related pain, neoplasm-related pain, opioid analgesics, opioids, pain

## Abstract

**Background:**

Opioids are a key component of pain management among patients with metastatic cancer pain. However, the evidence base available to guide opioid-related decision-making in individuals with advanced cancer is limited. Patients with advanced cancer or cancer that is unlikely to be cured frequently experience pain. Opioids are a key component of pain management among patients with metastatic cancer pain. Many individuals with advanced cancer are now living long enough to experience opioid-related harm. Emerging evidence from chronic noncancer pain literature suggests that longer-term opioid therapy may have limited benefits for pain and function, and opioid-related harms are also a major concern. However, whether these benefits and harms of opioids apply to patients with cancer-related pain is unknown.

**Objective:**

This manuscript outlines the protocol for the “Opioid Therapy for Pain in Individuals With Metastatic Cancer: The Benefits, Harms, and Stakeholder Perspectives (BEST) Study.” The study aims to better understand opioid decision-making in patients with advanced cancer, along with opioid benefits and harms, through prospective examination of patients’ pain experiences and opioid side effects and understanding the decision-making by patients, care partners, and clinicians.

**Methods:**

This is a multicenter, prospective cohort study that aims to enroll 630 patients with advanced cancer, 20 care partners, and 20 clinicians (670 total participants). Patient participants must have an advanced solid cancer diagnosis, defined by the American Cancer Society as cancer that is unlikely to be cured. We will recruit patient participants within 12 weeks after diagnosis so that we can understand opioid benefits, harms, and perspectives on opioid decision-making throughout the course of their advanced cancer (up to 2 years). We will also specifically elicit information regarding long-term opioid use (ie, opioids for ≥90 consecutive days) and exclude patients on long-term opioid therapy before an advanced cancer diagnosis. Lived-experience perspectives related to opioid use in those with advanced cancer will be captured by qualitative interviews with a subset of patients, clinicians, and care partners. Our data collection will be grounded in a behavioral decision research approach that will allow us to develop future interventions to inform opioid-related decision-making for patients with metastatic cancer.

**Results:**

Data collection began in October 2022 and is anticipated to end by November 2024.

**Conclusions:**

Upon successful execution of our study protocol, we anticipate the development of a comprehensive evidence base on opioid therapy in individuals with advanced cancer guided by the behavioral decision research framework. The information gained from this study will be used to guide interventions to facilitate opioid decisions among patients, clinicians, and care partners. Given the limited evidence base about opioid therapy in people with cancer, we envision this study will have significant real-world implications for cancer-related pain management and opioid-related clinical decision-making.

**International Registered Report Identifier (IRRID):**

DERR1-10.2196/54953

## Introduction

Patients with advanced cancer or cancer that is unlikely to be cured frequently experience pain [1,2]. Although the overall prevalence of pain and pain severity related to cancer appear to be decreasing, 40% of patients with cancer experienced pain during and up to 3 months after cancer treatment [1,3,4]. When stratified by cancer severity, patients with more advanced disease have higher rates of pain: up to 66%, compared to 39% for patients with localized or curative disease [4]. Although patients with any cancer can experience pain, those with breast, lung, head, and neck cancer experience pain most often [5]. Under- and untreated pain in individuals with cancer is associated with a variety of adverse health consequences, including functional limitations (eg, inability to work), suboptimal health behaviors (eg, reduced physical activity), emotional distress, social isolation, high health care use (eg, emergency department and inpatient admissions), and earlier death [6-9].

Opioids are a key component of pain management among patients with metastatic cancer pain [10]. Although rates of opioid prescribing in advanced cancer have not been well described, people with advanced cancer are prescribed long-term opioid therapy (ie, opioid prescription for at least 90 consecutive days) more often than people with limited-stage disease (66% vs 40%) [11]. A recent study using data from the National Survey on Drug Use and Health found that approximately half of cancer survivors with a recent diagnosis (within 12 months of the National Survey on Drug Use and Health survey) were prescribed an opioid during that year (54%) [12]. National guidelines from the American Society of Clinical Oncology [10] and the National Comprehensive Cancer Network [13] support opioids as a cornerstone of pain management for individuals with advanced cancer, suggesting that the benefits of opioid use outweigh the harms for this patient group.

However, many individuals with advanced cancer are now living long enough to experience opioid-related harm. Indeed, some advanced cancers are considered chronic diseases as patients are surviving longer due to improvements in cancer treatments [14]. For example, the median survival of individuals with metastatic breast cancer in a large national cohort of patients with breast cancer in France was 37 months [15], which has increased steadily over the previous decades [16]. Approximately one-third of individuals with metastatic breast cancer [17] or metastatic prostate cancer [18] survive for at least 5 years. This presents a complex clinical context for treating cancer-related pain with opioids, increasing the need to balance opioid-related benefits and harms.

Emerging evidence from the chronic noncancer pain literature suggests longer-term opioid therapy may have limited benefits for pain and function. For example, a recent meta-analysis of randomized controlled trials of opioids versus placebo for chronic (>3 month) “noncancer” pain observed a small improvement in pain of 0.69 (95% CI 0.56-0.82) on a scale of 0-10 that was less than the prespecified minimum clinically important difference of 1 [19]. Other systematic reviews and meta-analyses have yielded similar findings, including a lack of functional improvement with opioid therapy over placebo [20,21]. Analogous data do not yet exist for patients with advanced cancer; given the unique features of cancer pain and the commonly concurrent noncancer pain experienced by patients with cancer, it is important to better understand pain and pain management in persons with advanced cancer.

Opioid-related harms are also a major concern among people prescribed opioids. In one meta-analysis of 26 studies, 23% of participants decided to discontinue opioids due to side effects (eg, nausea and dizziness) [22]. In the general population, long-term opioid therapy is also associated with more serious harms; 21% to 29% develop opioid misuse, and 8% to 12% may progress to an opioid use disorder. Although more rare, opioid overdoses can occur (256 per 100,000 person-years among people recently prescribed opioids vs 36 per 100,000 years among those not prescribed opioids) [23,24]. In studies of noncancer pain, opioid-related harms are consistently related to both ingestion of high-dose opioids (ie, >90 mg morphine equivalents) and coprescription of sedating medications (benzodiazepines and gabapentin) [25-28]. People with cancer are more likely to be represented in the high-dose opioid groups and frequently experience polypharmacy [29,30]. However, whether these benefits and harms of opioids apply to patients with cancer-related pain is unknown.

This manuscript describes the study protocol for “Opioid Therapy for Pain in Individuals With Metastatic Cancer: The Benefits, Harms, and Stakeholder Perspectives (BEST) Study.” The study aims to better understand opioid decision-making in patients with advanced cancer, along with opioid benefits and harms, through prospective examination of patients’ pain experiences and opioid side effects and understanding the decision-making by patients, care partners, and clinicians.

## Methods

### Study Design

This is a multicenter, prospective cohort study that aims to enroll 630 patients with advanced cancer, 20 care partners, and 20 clinicians (670 total participants). The data collection is underway at 4 clinical sites. Site selection considered geographic diversity (Northeast, mid-Atlantic, West, and Southeast), the balance of urban versus rural patients, sufficient patient volume to reach the enrollment goal, and the ability to recruit based on previous success in cancer studies. Given well-established health disparities in cancer pain and its treatment, we selected sites that demonstrated a track record of successful recruitment of Black and Hispanic patient participants [31]. None of the chosen sites have opioid stewardship committees or programs, which could have the unintended consequence of limiting opioid prescribing [32]. Additionally, we conducted a comprehensive review of state opioid laws in preparation for the study [33], with most states specifying blanket cancer exemption for any opioid limitation, such as dose or limited day supply.

### Participants

Patient participants must have an advanced solid cancer diagnosis, defined by the American Cancer Society as cancer that is unlikely to be cured [2]. An advanced cancer diagnosis can include patients who have distant metastases or a recurrence. Patient participants must be their own decision maker as determined by the electronic medical record (EMR) and enroll within 12 weeks of their advanced cancer diagnosis date. The research team will confirm an advanced cancer diagnosis using medical record documentation of pathology results or radiology and oncology documentation. We will recruit patient participants early after diagnosis so that we can understand opioid benefits, harms, and perspectives on opioid decision-making throughout the course of their advanced cancer (up to 2 years). Lived-experience perspectives related to opioid use in those with advanced cancer will be captured by qualitative interviews with a subset of enrolled patients, clinicians, and care partners.

We will specifically elicit information regarding long-term opioid use (ie, opioids for ≥90 consecutive days) and exclude patients on long-term opioid therapy before an advanced cancer diagnosis. Consistent with previous studies, we confirm opioid use in the medical record and will ask patient participants, “Did you take a strong prescription pain medication known as an opioid or narcotic for at least 90 days in a row during the past year? Examples of opioids include oxycodone, hydrocodone, hydromorphone, morphine, fentanyl, buprenorphine, methadone, and combination products such as oxycodone/acetaminophen.” This question is consistent with other commonly used research definitions of long-term or chronic opioid therapy [34,35]. Notably, this long-term opioid use definition also excludes individuals who are receiving methadone or buprenorphine/naloxone (suboxone) for the treatment of opioid use disorder, as the potential benefits and harms of opioid therapy in this population are unique and merit separate investigation [36]. This will allow us to understand the benefits, risk factors for harm, and perspectives on opioid decision-making when a patient with newly diagnosed advanced cancer begins an opioid or continues a newly initiated opioid.

From the larger cohort, we will recruit 40 patient participants with advanced cancer—20 who are prescribed opioids at the time of enrollment and 20 who are not. From that sample of 40 patients, we will aim to obtain consent from care partners of at least 20 patients and clinicians of at least 20 patients.

We will exclude individuals younger than 18 years of age, prisoners, pregnant people, and patients currently enrolled in hospice at the time of recruitment from the study. Patient participants do not need to report pain at the time of diagnosis since pain can occur at any point in the cancer trajectory. By including patient participants without pain at the time of diagnosis of metastatic disease, the study has the added benefit of describing the natural history of pain in patients with metastatic cancer for 2 years after diagnosis, filling an existing literature gap [3]. Patients who meet advanced cancer eligibility criteria will be recruited irrespective of their predicted prognosis, due to the inherent challenges of predicting prognosis [37,38].

For qualitative interviews, care partner participants must be a spouse, partner, child, relative, or friend who helps the patient with activities of daily living and health care needs at home, consistent with the National Cancer Institute definition of family caregivers [39]. Clinician participants will be physicians or advanced practice clinicians caring for a patient participant who has opioid prescribing authority and is willing to participate in a qualitative interview.

### Theoretical Framework

**Figure 1:**
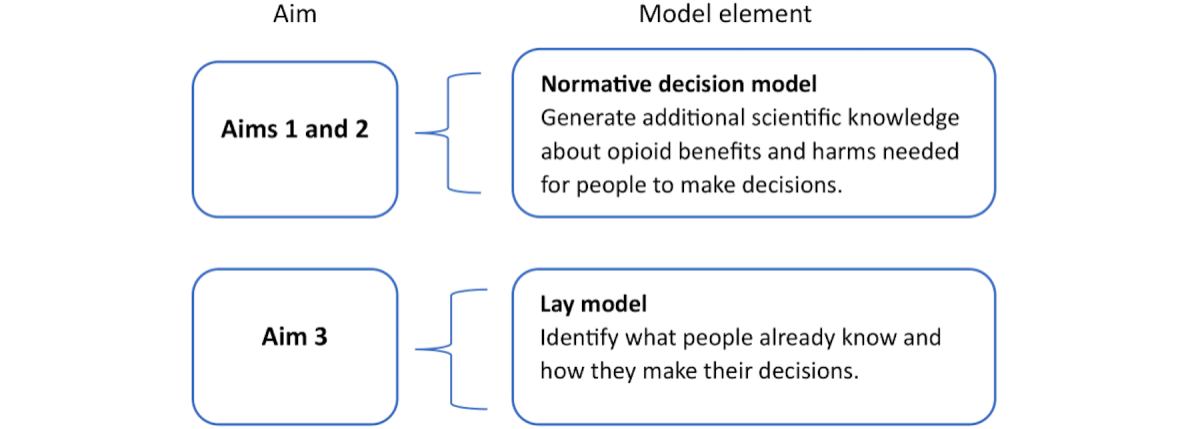
Study aims mapped to behavioral decision-making model element.

Our data collection will be grounded in a behavioral decision research (BDR) approach [40] that will allow us to develop future interventions to inform opioid-related decision-making for patients with metastatic cancer. Opioid-related decision-making refers to decisions to initiate opioids or continue opioids over time. The BDR approach consists of 2 components. The first is the characterization of a “normative decision model,” which describes the information distilled from existing scientific evidence that experts in the field believe that decision makers (patients, care partners, and clinicians) need to know to be able to make an informed decision. The second is a lay “mental model” of the decision, or what interested parties (ie, patients and care partners) already know and how they currently make their decisions. The normative decision model is a well-established approach for medical decision-making [41], including the development of opioid guidelines [42,43]. Consistent with previous studies, the lay decision model solicits perspectives on what patients, care partners, and clinicians consider foundational knowledge and how they make their decisions [44]. Interventions to support optimal decision-making can bridge the gap between the normative model and the contextual reality of how individuals are currently making those choices. Figure 1 provides the BDR framework-based approach mapped to the study aims.

### Specific Aims

Using the BDR framework, the objective of this study is to create evidence to guide opioid prescribing in patients with advanced cancer.

The study’s aims are as follows:

- Aim 1: To investigate the relationship between opioid therapy and opioid-related benefits.
- Hypothesis 1a: Prescribed opioids will be associated with decreased pain severity and pain interference (coprimary outcomes).
- Aim 2: To investigate risk factors for opioid-related harms.
- Hypothesis 2a: Certain coprescribed medications will be associated with an increased risk of opioid side effects (eg, benzodiazepines and somnolence).
- Hypothesis 2b: Younger age, history of substance use disorder, and history of mood disorders will be associated with a greater risk of opioid misuse and use disorder.
- Aim 3: To understand patient, care partners, and clinician perspectives on opioid-related decision-making.

### Study Procedures

#### Recruitment

Potential patient participants will be identified using EMRs; *International Classification of Diseases, Tenth Revision* diagnosis codes; direct clinician referral; and referral from informational handouts in patient-facing platforms (eg, clinic rooms and chemotherapy teach-back packets). Study sites will develop reports to generate lists of potentially eligible patient participants in accordance with all rules of the HIPAA (Health Insurance Portability and Accountability Act), preparatory to the research exception [45]. The research coordinator will review listed patients’ EMRs to verify eligibility following institution-specific procedures for contacting potential patient participants in clinic, over the telephone, or through letters. After receiving permission using one of the methods described above, trained research staff at each site will contact potential patient participants in-clinic or over the telephone to confirm eligibility. Subsequently, research staff will obtain consent, and data will be collected using Research Electronic Data Capture (REDCap; Vanderbilt University), a free and secure web-based application to capture research data [46].

#### Assessments

Once patients have consented and enrolled in the study, data collection will include patient-reported outcomes (PROs), chart reviews, and interviews. Table 1 provides an overview of the study procedures and time frame.

**Table 1:** Schedule of research activities.

Study stage	Screening and enrollment	Follow-up			
	Within 12 weeks of the date of diagnosis	Weekly after baseline	Monthly after baseline	Quarterly after baseline	Semiannually after baseline
Prescreening	✓				
Consent, screening, and contact information	✓				
Demographics and medical history	✓				
Baseline and demographics	✓				
Weekly 3-questions pain assessment: pain severity and inference		✓			
Monthly PRO^a^ assessment: mood, substance use, symptoms, and opioid misuse (Carey 2-year index)			✓		
Chart reviews: substance use, opioid misuse, and opioid overdose				✓	
Patient calls and chart review: Opioid prescription or dose, benzodiazepine prescription, over or under consumption of opioids					✓
TAPS^b^: substance use and opioid use disorder				✓	
Patient participants, care partners, and clinician interviews				✓	

^a^PRO: patient-reported outcome.

^b^TAPS: Tobacco, Alcohol, Prescription Medication, and Other Substance Use Tool.

Study staff will confirm patient demographics and cancer type from the medical record. Sites will use a chart abstraction tool to gather medication-related data every 6 months. Opioid dose will also be assessed through self-report by reading back the dose and instructions on the opioid bottle and confirmed in the EMR. Patient participants will consent to receiving a phone call from the study staff for pill counts during the latter part of the month of their prescriptions every 3 months.

Patient participants will complete baseline assessments and PROs electronically using REDCap, over the phone, or during clinic visits through a tablet by the study staff. If patient participants are unable to complete PROs, care partners will be permitted to complete the PRO assessments on behalf of the patient participants. We acknowledge that based on systematic reviews in oncology, there may be differences in patient-proxy reports, with proxies having a more negative view of the patient’s well-being [47].

The outcome measure for pain will be the Pain, Enjoyment, and General Activity (PEG) scale, a 3-item commonly reliable and validated measure used in oncology populations that asks about pain severity, pain interference in enjoyment of life, and pain interference in general well-being [48-50]. The PEG meets the Initiative on Methods, Measurement, and Pain Assessment in Clinical Trials recommendations and has a 7-day recall period [51]. The PEG will be collected weekly to provide longitudinal impact on pain severity, pain interference, and whether opioids precede any change in pain.

Additional monthly PROs will include information on mood (Functional Assessment of Cancer Therapy-General Well-Being Subscale); comorbidities (Self-Administered Comorbidity Questionnaire); functioning (Carey index); and if prescribed opioids, information on opioid misuse (Patient-Reported Outcomes Measurement Information System), opioid side effects (Patient-Reported Outcomes version of the Common Terminology Criteria for Adverse Events), and substance use (Tobacco, Alcohol, Prescription Medication, and Other Substance Use Tool) [52-56]. These measures were selected because of their favorable psychometrics in the study population, brevity, and similar recall periods. This patient-participant burden is similar to other studies among patients with metastatic cancer [57].

For Aim 3, we will conduct one-on-one interviews with patients, their care partners, and clinicians with the goal of better understanding opioid-related decision-making. These interviews will occur every 3 months for 2 years or until death. The rationale for this frequency is that it strikes a balance between interviewing people often enough for them to remember aspects of opioid decision-making since the last interview but not so often as to ask about opioid-related decisions more frequently than those decisions occur or cause undue participant burden.

### Analytic Plan

#### Aim 1: Data Analysis

This analysis will draw from all patient participants, regardless of whether they are prescribed opioids, so we can compare those who are with those who are not prescribed opioids. We will estimate differences in pain severity and interference between patients treated with opioids versus patients not treated with opioids using linear mixed-effects models, which will allow us to (1) account for repeated measures over time for each patient and (2) adjust for potential confounders. In the linear mixed-effects models, the dependent variables will be based on the PEG: pain severity (first question is a continuous 0-10 value) and pain interference (mean of 3 PEG items) at each time point. Current opioid prescription, baseline pain severity or interference, and other confounders (ie, factors statistically associated with the exposure and outcome) will be included as fixed effects, while the subject will be included as a random effect.

#### Power Calculation for Aim 1

Power calculations were performed by simulation to allow a range of parameters to vary in all scenarios. Unlike a randomized trial, in this study we will not have control over the ratio of patients allocated to the 2 comparison groups (eg, there will not be precisely a 1:1 ratio of patients prescribed opioids vs patients not prescribed opioids for comparison). Scenarios were considered where the proportion of patients prescribed opioids was allowed to vary from 30% up to 70%, all assuming a mean baseline PEG of 6 in the patients not prescribed opioids, a reduction in the PEG of 1 point for patients that are prescribed opioids, and a common standard deviation in PEG scores of 2 points [51]. All scenarios considered had at least 90% power to detect a difference of 1 point between groups with a total sample size of 630 patients (allowing for 30%-70% of the population to be prescribe opioids, meaning that with a range of 189 up to 441 patients prescribed opioids during the study period, there is ample power to detect differences between groups). All scenarios had at least 99% power to detect a 2-point difference on the PEG. We acknowledge that up to 40% of patients may drop out during the study due to the morbidity and mortality of patients in palliative care [58]. Data up until the point of attrition will be included in the analysis, and this has also been accounted for in our calculations.

#### Missing Data for Aim 1

In general, linear mixed-effects models provide unbiased estimates when the data are assumed to be missing at random, meaning that the likelihood of a missing value is not related to the values of the outcome data (in this case, the assumption that missing values for pain are not related to the severity of the patient’s pain). The setting of this study will have two basic missingness problems: (1) data that are missing while the patient still lives, either because the survey could not be completed or the patient did not wish to complete the pain instrument; and (2) pain data that are missing because the patient is deceased. The missing at random assumption can never be fully confirmed, so we will perform three sensitivity analyses to see how our results vary under different approaches to handling the missing pain values: (1) multiple imputations; (2) imputing the worst pain value for patients that fail to complete the pain instrument for any reason (including death before the scheduled assessment); and (3) imputing the worst pain value for patients that fail to complete the pain instrument, but omitting patients that have died. It should be noted that in this patient population, death is an expected outcome and not necessarily a negative outcome with respect to end-of-life suffering and pain management.

#### Aim 2a: Data Analysis

To test our hypothesis that coprescribed medications will be associated with an increased risk of opioid side effects (eg, benzodiazepines and somnolence), we will include only study patient participants who are prescribed opioids. Linear mixed-effects models will be used to assess the relationships between selected risk factors and the corresponding suspected opioid side effects. Each side effect is scored on the respective weekly or monthly assessments. Analyses will be conducted to examine the relationship between coprescribed medications and each of the respective opioid side effects (constipation, nausea, dry mouth, and somnolence) as well as targeted relationships specifically of interest for individual exposures or side effects combinations, including (1) coprescription of other constipating medications and constipation, (2) coprescription of other constipating medications and nausea, (3) coprescription of anticholinergic medications and dry mouth, and (4) coprescription of benzodiazepines with somnolence. We will also examine the association between which opioid is prescribed (ie, morphine vs oxycodone) and each of the same respective opioid side effects (constipation, nausea, dry mouth, and somnolence). The use of linear mixed-effects models will provide estimates of the effect of each risk factor of interest on each of the respective side effects while also allowing the inclusion of longitudinal measurements from patient participants.

#### Power Calculation for Aim 2a

With an overall sample size of 630, we anticipate approximately 50% (315/630) of the study sample to be prescribed opioids, leaving 315 patient participants available for the analyses pertinent to this hypothesis. We examined a range of scenarios allowing anywhere from 10% to 30% of the eligible participants to have each exposure, performing power analyses to detect a 0.5-point difference in means for each side effect on the 1-5 scale (assuming a SD of 1 point). Power is about 65% if the exposure is rare (10% of patients prescribed opioids) but increases to 87% if the exposure is present in 20% of patients prescribed opioids and 93% if exposure is present in 30% of patients prescribed opioids. Again, we account for up to 40% attrition during the study. Note that patient participants will contribute data until they drop out, and this data will be included in the analysis.

#### Aim 2: Data Analysis to Test Hypothesis 2b

To test our hypothesis that younger age, history of substance use disorder, and history of mood disorders will be associated with a greater risk of opioid misuse and use disorder, we will perform multivariable Cox regression analyses to assess the respective risk factors of interest. We will use a single prespecified model that includes age, history of substance use disorder, and history of mood disorders using baseline PRO data and chart review.

#### Power Calculation for Aim 2b

With a total sample size of 630 patients recruited in Aim 1, the power calculation for this subaim assumed a scenario where approximately 50% (315/630) of the patients are prescribed opioids, leaving 315 patients available for this aim. We anticipate opioid misuse will occur in 20% to 30% of patients. For a rarer event such as opioid use disorder, which is estimated to occur in approximately 10% of the patients, a sample size of 315 patients will be sufficient to support a model with 5 candidate predictor variables.

#### Missing Data for Aim 2

Unlike Aim 1, missing data should not affect analyses in this aim. The development of opioid-related complications will also be considered an event if it is known to occur and otherwise assumed not to occur during the period patients are under observation.

#### Aim 3: Data Analysis

The purpose of Aim 3 is to identify what patients, care partners, and clinicians already know and how they make opioid-related decisions to develop the lay model of the BDR. To determine decisional influences, the BDR framework uses the “mental models approach” to elicit these views [59]. To create the BDR framework lay model, we will structure the patient and care partner interviews by focusing on (1) what they already know about opioids, (2) how they make decisions, (3) examining whether this is consistent with or contrary to the normative model of evaluating risks and harms, and (4) how they think decisions around opioid use should ideally be made. The interviews begin with general questions and then become more specific, asking about each topic from the expert model. We will structure the clinician interviews by focusing on (1) their assessment of the patient’s pain; (2) approaches to best treating it, including opioids; and (3) their decision-making around opioid prescribing. We will also evaluate whether different patient, care partner, and clinician perspectives are consistent with or contrary to one another.

For the semistructured interviews (40 participants with metastatic cancer from our larger cohort, 20 who are prescribed opioids at the time of enrollment, and 20 who are not), we aim to understand perspectives on deciding to initiate or continue opioid therapy or not. This sample size is based on evidence that suggests thematic saturation is achieved at 8-16 interviews; since we expect up to 40% attrition, this approach will ensure we have at least 20 participants [60]. We will purposively sample [61] to ensure diversity on race, site, and baseline PEG value; we will also recruit some individuals who have a history of a substance use disorder and are therefore at particularly high risk for negative consequences when prescribed opioids.

Similarly, we will recruit patient participants’ care partners and clinicians. We will aim for at least 10 people who have care partners who consent to the group prescribed opioids at enrollment and 10 care partners of patient participants who have been prescribed opioids at enrollment. This approach will ensure that we have enough care partners to provide adequate perspective but not severely bias our sample by only allowing the enrollment of individuals who have care partners willing and able to enroll in this study. As described above, we will also purposefully sample 25% of patient participants who do not have care partners so that these perspectives are represented. Interviewing patients and their care partners s and clinicians will allow for triangulation of findings. Additionally, following patients, care partners, and clinicians longitudinally will allow for a detailed understanding of how decision-making changes over the course of metastatic cancer, allow participants to reflect on what they would have liked to know earlier based on what they know now, and provide real-time data on actual decisions (eg, initiation or discontinuation of opioids) on which participants can be asked to comment. Given the challenges inherent in engaging clinicians, we will aim for 15-minute focused interviews.

Trained qualitative researchers will collect and analyze the qualitative data. Interviews will be conducted remotely (through phone or Zoom [Zoom Video Communications], according to the interviewee’s preference), and interview recordings will be transcribed verbatim (with details that might identify the interviewee redacted). Coding the data will require a hybrid deductive-inductive approach. A prespecified set of codes will be identified from the normative model. The presence and absence of these codes (eg, the accuracy of knowledge about the relationship between opioids and addiction) will be used to document the consistency between the normative model and stakeholder perspectives. In addition, we will take an inductive analytical approach to identify emergent values and beliefs that are not present in the normative models. Codes will be developed through open coding of the transcripts to determine topics and themes that emerged in the interview transcripts with input on topics or themes that the study team anticipates being relevant, resulting in simultaneously inductive and deductive development of the codebook. A draft codebook including detailed code definitions will be discussed with the study team to ensure that codes reflect both the data as they are emerging as well as relevant topical themes that are important to the team. Codes will be thoroughly checked to determine that definitions are distinct enough to reduce ambiguity in the coding process.

About 25% of the transcripts will be coded by 2 independent, trained qualitative coders to ensure quality and consistency in coding. Coding will then be compared for the purposes of calculating Cohen kappa inter-coder reliability scores [62]. Any coding discrepancies identified during this comparison will be adjudicated by the coders until full agreement on coding is achieved. The finalized coding on these transcripts will be recorded in a single ATLAS.ti file, following which the primary coder will complete the coding of the remaining transcripts according to the codebook and through the adjudication process and discussion by the study team with content and methodologic expertise. This process allows for consistency and quality across transcripts. Once coding is complete and uploaded to ATLAS.ti with quotes associated with codes, we will thematically analyze and determine the relative frequencies of various codes and the most salient themes. The resulting thematic analysis will be discussed with the entire investigator team to use the findings to build lay models of patient, care partner, or clinician decision-making around opioid use for patients with metastatic cancer.

### Ethical Considerations

This study has been reviewed and approved by the University of Pittsburgh Institutional Review Board (STUDY20090231), which serves as the institutional review board of record for all research locations. Informed consent is obtained by trained research staff members at each site. The study was granted a waiver of HIPAA authorization to identify participants who meet study inclusion criteria. The study was granted a waiver to document informed consent for the qualitative interviews conducted in Aim 3. Verbal consent will still be obtained by interviewers before commencing the interview.

Study data will be identifiable while the study is ongoing, as there are many contact points with participants. All study data are segregated by research site, so recruitment team members only have access to the data for participants they have enrolled. The University of Pittsburgh team has access to all study records as the team conducts follow-up assessments. All results that will be published in this study in the future will be done in an aggregate, deidentified manner.

We will reimburse patient participants US $10 for the baseline questionnaire, US $2 per weekly questionnaire, and US $10 per monthly questionnaire (up to US $394 if all questionnaires are completed over the 2 years). Patient participants who complete at least 80% of questionnaires during each year of follow-up will be eligible for an annual bonus payment of US $20. This results in US $434 of total possible compensation for Aims 1 and 2. For Aim 3, patients and care partners will be reimbursed US $30 per interview. Clinicians will not be compensated.

## Results

This study was peer-reviewed and funded by the National Institute of Nursing Research in September 2021. Recruitment began in October 2022; we anticipate completing recruitment by November 2024. Follow-up assessments will end by November 2026, at which time we can finalize our data analysis.

## Discussion

This paper presents the study protocol for the benefits, harms, and stakeholder perspectives regarding opioid therapy for pain in individuals with metastatic cancers .The study also uses PRO measures, qualitative data collection, and repeated assessments to ascertain how decision-making differs throughout the cancer trajectory, including at the end of life. The protocol is innovative because it addresses the research gap on the benefits and harms of opioids in metastatic cancer in a novel way. This includes our prospective design in patients recently diagnosed with advanced cancer, the collection of data from multiple perspectives (patients, clinicians, and care partners), and the inclusion of a well-known decision-making framework that can facilitate the design of future interventions.

There are several potential challenges or limitations to the research protocol. The first is likely challenges of retention and recruitment that are expected in longitudinal studies, especially in individuals with cancer and serious illnesses. Second, there is a potential for low enrollment or a high rate of missing data due to death, illness, dropout, or participant burden. We have attempted to account for these anticipated issues by having backup recruitment sites and conservative sample size estimates. Third, opioid prescribing is an evolving area of clinical practice and subject to federal and state oversight. It is possible that local and state regulations or institutional policies will change during the study period, resulting in a decrease in opioid prescribing and recruitment issues. Last, prospective investigation of adverse effects such as overdose or opioid-related mortality may be difficult to capture because these events are relatively rare.

At the end of the study, we anticipate the development of a comprehensive evidence base on opioid therapy in individuals with advanced cancer, guided by the BDR framework. The information gained from this study will be used to guide interventions to facilitate opioid decisions among patients, clinicians, and care partners. Given the limited evidence base about opioid therapy in people with cancer, we envision this study will have significant real-world implications for cancer-related pain management and opioid-related clinical decision-making.

## Appendix

Multimedia Appendix 1 Peer-reviewer report from the National Institute of Nursing Research.

## Abbreviations

BDR: behavioral decision research
BEST: Benefits, Harms, and Stakeholder Perspectives
EMR: electronic medical record
HIPAA: Health Insurance Portability and Accountability Act
REDCap: Research Electronic Data Capture
PRO: patient-reported outcome
PEG: Pain, Enjoyment, and General Activity

## Data Availability

The data sets generated during and/or analyzed during this study are available from the corresponding author on reasonable request.
