# Supplementary material for: Benefits, Harms, and Stakeholder Perspectives Regarding Opioid Therapy for Pain in Individuals With Metastatic Cancer: Protocol for a Descriptive Cohort Study
Source: JMIR Res Protoc. 2024 Mar 13;13:e54953. doi: 10.2196/54953 (PMC10973954; doi:10.2196/54953)
Supplement: Multimedia Appendix 1 [file resprot_v13i1e54953_app1.pdf]

1R01NR020031-01 Merlin, Jessica

RESUME AND SUMMARY OF DISCUSSION: In this application the Investigators propose to develop interventions that inform opioid-related decision-making for patients with metastatic cancer using the Behavioral Decision Research (BDR) framework. The panel agreed that the proposed study is significant, and if successful, findings may have the potential to improve our knowledge about opioid benefits and harms and inform opioid-related decision-making in cancer patients. They also discussed numerous strengths including a strong investigative team with complementary expertise, a novel focus on patients with metastatic cancer, and an excellent environment. Some minor addressable weaknesses were discussed including lack of clarity about how confounding factors such as side effects will be handled, inadequate discussion about the sample heterogeneity regarding the type of metastatic cancers, pain and opioid use, lack of clarity about if the sample size of 630 participants is adequate, and limited discussion about potential attrition. Overall, the panel agreed that this is an excellent application and anticipates the findings to have a high impact on examining the risks and benefits of opioid therapy in patients with metastatic cancer.

DESCRIPTION (provided by applicant): Opioid therapy for pain in patients with metastatic cancer is a critical yet understudied area. Pain is experienced at some point by most patients with metastatic cancer. Prescribed opioids are a cornerstone of treating pain; the prevalent belief in the field has been that the benefits of palliating pain in metastatic cancer with opioids nearly always outweigh any potential harms. This approach to opioid-related decisions in patients with metastatic cancer implies that patients' prognoses are either so short that these harms are not meaningful, or that benefits of opioids are substantial while harms are relatively minimal. Research on the benefits and harms of opioid therapy has exploded in the past decade but primarily focuses on individuals with chronic "non-cancer" pain. However, patients with cancer have been routinely excluded from these studies and resulting recommendations that favor more conservative opioid prescribing. The few studies of patients with cancer generally support that serious harms occur, but have significant methodologic limitations. Additionally, there are unique considerations in individuals with metastatic cancer, including life-limiting disease that may last years, high pain rates, and opioids as accepted standard of care. Therefore, assessing benefits and risk factors for opioid-related harms in individuals with metastatic cancer is a critical gap in the literature and key to opioid-related decision-making. The long-term goal of this program of research is to develop interventions that inform opioid-related decision-making for patients with metastatic cancer. We will use the Behavioral Decision Research framework to create a comprehensive evidence base on which these interventions can be grounded, which is the objective of the present application. To accomplish our aims, we have partnered with the NINR- funded Palliative Care Research Cooperative to develop a prospective cohort of patients newly diagnosed with metastatic cancer. We propose the following Aims in patients with metastatic cancer: Aim 1: Investigate the relationship between opioid therapy and opioid-related benefits. Hypothesis 1: Opioid therapy will be associated with decreased pain severity and pain interference (co-primary outcomes). Aim 2: Investigate risk factors for opioid-related harms. Hypothesis 2a: Certain co-prescribed medications will be associated with increased risk of opioid side effects (e.g., benzodiazepines and somnolence). Hypothesis 2b: Younger age, history of substance use disorder, and history of mood disorders will be associated with greater risk of opioid misuse and use disorder. Approach: We will use linear mixed effects models (2a) and time-to-event analyses (2b). Aim 3: Understand stakeholder (patient, family caregiver, clinician) perspectives on opioid-related decision-making. Completion of these aims will lead directly to an R-series proposal to develop and test a novel intervention to inform opioid decision-making, followed by R-series proposals to study intervention effectiveness and implementation. This work has the potential to transform opioid prescribing and pain management for patients with metastatic cancer.

PUBLIC HEALTH RELEVANCE: By investigating opioid-related benefits and risk factors for harms in individuals with metastatic cancer, the proposed study will provide evidence that patients, family caregivers, and clinicians need when engaged in opioid-related decision-making. This has the potential to transform pain and opioid care for patients with metastatic cancer, and as such, is directly responsive to National Institute of Nursing Research priorities to improve the health of individuals with serious illness and the National Cancer Institute's mission of helping people with cancer lead longer, healthier lives.

CRITIQUE 1

Significance: 1
Investigator(s): 1
Innovation: 2
Approach: 2
Environment: 1

Overall Impact: This is a prospective study of opioid therapy and related benefits and risk factors for harms in patients with metastatic cancer, coupled with qualitative interviews to understand patient, caregiver, and clinicians' perspectives on opioid-related decision making. The proposed study fills an important gap regarding evidence-base for opioid treatment in cancer patients, who are often excluded from studies of opioids despite the weak evidence base for current practice. The study has a high likelihood of advancing understanding of opioid-related benefits and harms, and of laying the groundwork for future studies testing interventions to inform opioid decision making. The investigators have designed an approach to minimize the challenges of researching this question where a randomized trial providing or withholding opioids would be ethically problematic and have defined inclusion criteria and thoughtfully selected sites to maximize the real-world applicability of their findings. While there remain limitations in the approach regarding potential for confounding by indication and concerns that recruitment and retention may prove challenging, these were appropriately addressed in the narrative and were minimal in contrast to the numerous strengths in the team, innovation, and approach.

1. Significance:

Strengths

- Addresses an important gap in the literature, that of benefits vs harms of opioids in the treatment of metastatic cancer pain.
- Considers role of medications in addition to opioids, as practically speaking pain treatment often involves a number of medications/medication trials and adjustments.
- Approach anticipates future investigations that have good likelihood of building on findings and leading to improvements in patient treatment.

Weaknesses

- None identified.

2. Investigator(s):

Strengths

- Experienced investigators with good complement of expertise.
- Interprofessional collaboration is a strength.

Weaknesses

- None noted by reviewer.

3. Innovation:

Strengths

- Focuses on an important and understudied area; work on opioids has by and large excluded this patient population, leading to gaps in knowledge. (Major)
- Takes a thoughtful approach to a prospective study to collect information in an area where randomization or withholding of usual care would be ethically problematic; this pragmatic approach with a focus on yielding generalizable and actionable information is innovative. (Major)

Weaknesses

- Weakness relevant to innovation pertain to the approach, as the prospective study design and general inclusiveness may not adequately address confounding by indication. (moderate)

4. Approach:

Strengths

- The investigators take a pragmatic approach to investigating relevant questions within the constraints posed by the current care standards, condition, and population. They have relatively permissive inclusion criteria and will collect information on pain and other outcomes prospectively with patients exposed to care as usual. (Major)
- The design is appropriate to the questions being asked, and the considerations around site selection, recruitment, and retention reflect a nuanced understanding of the issues under investigation.
- The Behavioral Decision Research Framework is well-suited, and study aims especially around lay model development appear well thought out.

Weaknesses

- The study patients will likely be experiencing health changes and potentially side effects of medications in additional to opioids; attributing benefits and harms to opioids may be challenging. (moderate)

5. Environment:

Strengths

- The environment is very strong and the selection of multiple sites with differing characteristics and patient populations is well thought out and described. (Major)

Weaknesses

- Multiple sites and somewhat complex design may make management of the project complicated. (minor)

Protections for Human Subjects:
Acceptable Risks and/or Adequate Protections

- The risks are well-described and protection from risk appropriate.

Data and Safety Monitoring Plan (Applicable for Clinical Trials Only):
Acceptable

Inclusion Plans:

- Sex/Gender: Distribution justified scientifically
- Race/Ethnicity: Distribution justified scientifically
- For NIH-Defined Phase III trials, Plans for valid design and analysis: Not applicable
- Inclusion/Exclusion Based on Age: Distribution justified scientifically

Vertebrate Animals:
Not Applicable (No Vertebrate Animals)

Biohazards:
Not Applicable (No Biohazards)

Applications from Foreign Organizations:
Not Applicable (No Foreign Organizations)

Select Agents:
Not Applicable (No Select Agents)

Resource Sharing Plans:
Not Applicable (No Relevant Resources)

Authentication of Key Biological and/or Chemical Resources:
Not Applicable (No Relevant Resources)

Budget and Period of Support:
Recommended budget modifications or possible overlap identified:

CRITIQUE 2

Significance: 1
Investigator(s): 1
Innovation: 2

Approach: 5
Environment: 1

Overall Impact: This project aims to create an evidence base for interventions to optimize opioid prescribing for patients with metastatic cancer by tracking pain relief, side effects, and the onset of opioid misuse and use disorder among patients followed prospectively, with the primary exposure being use of opioid pain medications. It will also investigate perspectives on opioid-related decision-making among key stakeholders (patients, clinicians, family members). The proposal has the potential to make a substantial impact on efforts to improve pain control and health outcomes on cancer patients, who are often overlooked in policy efforts focused on opioids. Key strengths of the proposal include a large and diverse multi-site recruitment strategy, a comprehensive set of measures and outcomes, and the integration of mixed methods. The main limitation relates to the possible presence of residual confounders, including confounding by indication due to unmeasured severity, lack of attention to the use of alternative pain control therapies, and unmeasured motivations that differ between patients who accept versus reject opioid therapy. Overall, the impact score is driven by the clear potential of the study to create new knowledge to improve opioid prescribing among patients with metastatic cancer, tempered by concerns about the study approach.

1. Significance:

Strengths

- Opioid use disorder and overdose is a national public health crisis, and there are widespread efforts to improve opioid pain reliever prescribing, but these efforts often overlook patients with metastatic cancer. The investigators note that patients with metastatic cancer often survive for long enough to experience the adverse consequences of chronic opioid use and it is unclear how much pain relief is accomplished through chronic opioid therapy. The conceptual framework that encompasses both normative and lay decision models for opioid prescribing provide a clear foundation for addressing patient preferences and objective health outcomes in a single framework.

Weaknesses

- None noted by reviewer.

2. Investigator(s):

Strengths

- Dr. Merlin has clear expertise in both cancer treatment and opioid-related research and has an appropriate amount of responsibility in the project. The team of supporting investigators bring a wealth of complementary expertise in patient-reported outcomes, addiction medicine, cancer care, and biostatistics.

Weaknesses

- None noted by reviewer.

3. Innovation:

Strengths

- The integration of both qualitative stakeholder perceptions with patient-reported outcomes in the context of opioid treatment for metastatic cancer is a novel contribution to the field. The

comprehensive perspective that considers both potential benefits and harms of opioid prescribing is also innovative.

Weaknesses

- None noted by reviewer.

4. Approach:

Strengths

- The study has a strong multi-site recruitment plan and a good projected sample size. There is an integration of different relevant outcomes for Aims 1 and 2, including measures from patient reports, chart reviews, and pharmacy calls. The stakeholder interviews for Aim 3 have a good conceptual basis and will complement Aims 1 and 2. The prospective cohort design provides some ability to address confounding between and within subjects in the study.

Weaknesses

- Selection into opioid use is fundamentally a difficult process to measure, even after accounting for observable variables. The approach to consider and account for selection bias at baseline and across the study period has serious limitations. It seems like the policy variation that the investigators are trying to avoid (e.g., the presence of state laws that might limit access to opioids) actually would provide some helpful quasi-experimental variation. Even if this route is not pursued, some more explicit strategy to account for patient selection into treatment (including the clinician who writes the prescriptions and who often vary in their willingness to prescribe opioids) could be accounted for.
- Other variables that are likely to confound the study are illicit opioid use (e.g., non-prescribed opioids) and access to alternative pain control strategies (including physical therapy, acupuncture, or medical cannabis).
- Study attrition remains somewhat of a concern. The financial incentives might be optimized to increase remuneration for later in the study or scaled up to bring back people who have left the study.

5. Environment:

Strengths

- Very strong institutional environments across all study institutions.

Weaknesses

- None noted by reviewer.

Protections for Human Subjects:

Acceptable Risks and/or Adequate Protections

- No concerns

Data and Safety Monitoring Plan (Applicable for Clinical Trials Only):

Not Applicable (No Clinical Trials)

Inclusion Plans:

- Sex/Gender: Distribution justified scientifically
- Race/Ethnicity: Distribution justified scientifically
- For NIH-Defined Phase III trials, Plans for valid design and analysis: Not applicable
- Inclusion/Exclusion Based on Age: Distribution justified scientifically
- Not applicable.

Vertebrate Animals:
Not Applicable (No Vertebrate Animals)

Biohazards:
Not Applicable (No Biohazards)

Applications from Foreign Organizations:
Not Applicable (No Foreign Organizations)

Select Agents:
Not Applicable (No Select Agents)

Resource Sharing Plans:
Not Applicable (No Relevant Resources)

Authentication of Key Biological and/or Chemical Resources:
Not Applicable (No Relevant Resources)

Budget and Period of Support:
Recommend as Requested

CRITIQUE 3

Significance: 2
Investigator(s): 1
Innovation: 2
Approach: 6
Environment: 1

Overall Impact: This multi-institutional and multi-investigator proposal led by Dr. Merlin focuses on studying the risks and benefits of opioid therapy in patients with metastatic cancer. The team argues that patients with metastatic cancer traditionally get overlooked as being in need of opioid therapies and the potential risks and harms are overlooked in the process. In this R01 proposal, they will recruit a cohort of patients within a few weeks of a diagnosis of metastatic cancer and follow them to study pain

and other harms of opioids. They will compare those using and those not using opioids. The merits of this team and proposal are many. However, there are also some concerns. Of note, there is a lot of heterogeneity in terms of: use of opioids (it is not one-time and likely varies in terms of starting time, stopping, dose adjustments), indications for opioids, variability in self-reported pain (which is not balanced by randomization in this case and likely influenced by self-selection), type and location of cancers, etc. Given this much heterogeneity, it is hard to imagine that 630 participants will be sufficient to adequately examine these differences statistically speaking. Furthermore, the recruitment itself is ambitious and the scope and size of this multi-institution partnership throws the PI into a challenging management role. While the topic is hugely important, the methodological concerns outweigh the significance I would encourage the investigators to consider re-submitting their proposal, but attempting to address the heterogeneity by focusing the cohort on a few key types of metastatic cancers or finding other ways to lower the impact of heterogeneity on this otherwise very important area of work.

1. Significance:

Strengths

- This is an important and as yet unexplored area.

Weaknesses

- The heterogeneity of the issue at hand is extensive and would suggest that it will be challenging to study.

2. Investigator(s):

Strengths

- The PI is a rising star in Palliative Medicine and appears to have an impressive portfolio of funded grants, publications, and presentations of influence.
- The PI is supported by a team that includes a statistician (Althouse), measurement/metrics expert (Bennett), psychologist (Bulls), anthropologist (Hamm), behavioral economist (Krishnamurti), nurse ethicist (Meghani), and clinicians (Fischer, LeBlanc, Leibschutz, Richie, Smith, Temel).
- The Investigator team has the support of a palliative care nursing research center.

Weaknesses

- The team has a very large number of investigators from a variety of institutions and this can be unwieldy; it is not immediately clear that the PI has ever managed a team this large.

3. Innovation:

Strengths

- The focus on patients with metastatic cancer is unique and advances the field.
- The methods for collecting weekly and monthly data from participants are novel.

Weaknesses

- A prospective cohort study, while innovative, is a challenging proposition.
- The retention of such a cohort is especially challenging, but I am also concerned that the incentives for responding are small and that the responses may be influenced by knowing that patients are “being watched” and used to justify their own feelings about opioids.

4. Approach:

Strengths

- A prospective cohort of this group of patients would be a first.
- The weekly to monthly frequency of data collection is good.

Weaknesses

- The project is ambitious and to address the likely challenges in recruiting sufficient patients, the PI has chosen to partner with multiple institutions as recruiting sites for the study. From a study management perspective, this may be more challenging than described.
- Recruiting a cohort is a substantial task and it is not entirely clear that the investigator team has this expertise and experience.
- One of the challenges of this specific cohort is that their cohort has an especially high risk of mortality; is there any plan to "replenish" the cohort or recruit additional waves of participants? In the investigators' own descriptions, 2/3 of participants will die in 36-60 months.
- The comparison planned in aim 1 seems to assume that opioid prescription is a single-time, static event, when in fact, it is likely that this will change (on, off, higher doses, lower doses) over time and is more dynamic than it is being made out to be.
- Pain measurement is inherently subjective, and this reviewer remains less convinced that stoicism and/or lower thresholds for pain won't influence the results. Furthermore, unlike an RCT where this would at least be balanced by randomization, here, there are likely to be selection effects (both due to choices of clinicians and patients, and due to differences in indication). There are also selection or social desirability effects in how participants will respond.
- There is also heterogeneity in the type of metastatic cancers, which again increases the heterogeneity.
- What happens to those that go to hospice or have home hospice?
- In trying to count the opioid use, what about situations where there are additional prescribers (pain clinics, etc.) or fragmented care (different systems)?
- What about the influence of therapies – e.g., radiotherapies, chemotherapies, etc. and their influence on pain.
- Statistically, given all of the heterogeneity described above, the sample size will be wholly insufficient to answer the questions being posed.

5. Environment:

Strengths

- Institutions with adequate resources to achieve aims.

Weaknesses

- None noted by reviewer.

Protections for Human Subjects:

Acceptable Risks and/or Adequate Protections

Data and Safety Monitoring Plan (Applicable for Clinical Trials Only):
Not Applicable (No Clinical Trials)

Inclusion Plans:

- Sex/Gender: Distribution justified scientifically
- Race/Ethnicity: Distribution justified scientifically
- Inclusion/Exclusion Based on Age: Distribution justified scientifically

Vertebrate Animals:
Not Applicable (No Vertebrate Animals)

Biohazards:
Not Applicable (No Biohazards)

Applications from Foreign Organizations:
Not Applicable (No Foreign Organizations)

Select Agents:
Not Applicable (No Select Agents)

Resource Sharing Plans:
Not Applicable (No Relevant Resources)

Authentication of Key Biological and/or Chemical Resources:
Not Applicable (No Relevant Resources)

Budget and Period of Support:
Recommend as Requested

THE FOLLOWING SECTIONS WERE PREPARED BY THE SCIENTIFIC REVIEW OFFICER TO SUMMARIZE THE OUTCOME OF DISCUSSIONS OF THE REVIEW COMMITTEE, OR REVIEWERS' WRITTEN CRITIQUES, ON THE FOLLOWING ISSUES:

PROTECTION OF HUMAN SUBJECTS: ACCEPTABLE

INCLUSION OF WOMEN PLAN: ACCEPTABLE

INCLUSION OF MINORITIES PLAN: ACCEPTABLE

INCLUSION ACROSS THE LIFESPAN: ACCEPTABLE
